# Exploring the Benefits of Cranberries in Dentistry: A Comprehensive Review

**DOI:** 10.3390/biomedicines14010085

**Published:** 2025-12-31

**Authors:** Isabella Schönhofen Manso, Yasmim Guterres Bauer, Eduarda Blasi Magini, Gabriel Leonardo Magrin, Izabella Thais da Silva, Ariadne Cristiane Cabral Cruz

**Affiliations:** 1 Post-Graduation Program in Dentistry, Federal University of Santa Catarina, Florianópolis 88040-900, Brazil; 2 Laboratory of Applied Virology, Federal University of Santa Catarina, Florianópolis 88040-900, Brazil; 3 Post-Graduation Program in Pharmacy, Federal University of Santa Catarina, Florianópolis 88040-900, Brazil

**Keywords:** Cranberry, dentistry, review, health benefits, bioactive compounds

## Abstract

Objectives: Despite the increasing scientific evidence regarding the application of Cranberries in dentistry, a comprehensive understanding of their potential benefits, active constituents, and mechanisms of action remains lacking. Consequently, this narrative review aims to meticulously analyze and consolidate the existing scientific literature on the utilization of Cranberries for the prevention and treatment of oral diseases. Materials and Methods: Electronic databases (PubMed, Scopus, and Web of Science) were searched up to October 2025. This review included *in vitro*, *in vivo*, and clinical research studies. A two-phase selection process was carried out. In phase 1, two reviewers independently screened titles and abstracts to identify potentially eligible studies. In phase 2, the same reviewers performed the full-text assessments of the eligible articles. Results: Among the 93 eligible articles, most assessed Cranberry use in Cariology (n = 28) and Periodontics (n = 26). Biofilm and microbial virulence factors (n = 46) were the most frequently studied topics. Cranberry extract (n = 32) and high-molecular-weight non-dialyzable material (NDM) (n = 23) were the most evaluated Cranberry fractions. Overall, Cranberry-derived compounds were identified as non-toxic and demonstrated promising antimicrobial activity against dental caries-related microorganisms in preclinical studies (n = 20). Regarding periodontal and peri-implant diseases, Cranberry demonstrated host immune modulator effects, counteracting the inflammatory and destructive mechanisms (n = 8). Additionally, Cranberries presented benefits in reducing the inflammation associated with periodontal disease and temporal mandibular joint lesions (n = 1). Regarding dental erosion, Cranberry inhibited dentin erosion (n = 4); however, no effect was observed on enamel lesions (n = 2). As an antioxidant agent, Cranberry showed effectiveness in preventing dental erosion (n = 18). Beyond that, Cranberry neutralized reactive oxygen species generated immediately after dental bleaching, enhancing bond strength (n = 2) and counteracting the oxygen ions formed on the tooth surface following bleaching procedures (n = 3). In osteoclastogenesis assays, A-type proanthocyanidins inhibited bone resorption (n = 1). In osteogenic analysis, preservation of hydroxycarbonate apatite deposition and an increase in early and late osteogenic markers were observed (n = 2). Conclusions: Cranberry bioactive compounds, both individually and synergistically, exhibit substantial potential for diverse applications within dentistry, particularly in the prevention and management of oral and maxillofacial diseases. This review provides insights into the plausible incorporation of Cranberries in contemporary dentistry, offering readers an informed perspective on their potential role.

## 1. Introduction

Cranberries have garnered widespread recognition for their considerable potential in promoting human health [1,2,3,4]. *Vaccinium macrocarpon*, *Vaccinium oxycoccus*, and *Vaccinium microcarpum* are species of Cranberry, with *Vaccinium macrocarpon* being the most extensively studied due to its distinctive phytochemical profile and bioactivity [5,6,7]. Historically, Cranberries have been used since the 17th century as a medical fruit to manage blood disorders, lipid and glucose metabolism, hepatic steatosis, and even cancer [8,9,10,11,12,13,14]. More recently, Cranberries have become best known for their efficacy in preventing urinary tract infections, which has been largely attributed to the presence of A-type proanthocyanidins capable of inhibiting bacterial adhesion to epithelial cells [2,14]. The broad spectrum of biological activities observed in Cranberry arises from its complex polyphenol composition, which includes compounds with anti-bacterial, anti-adhesive, anti-inflammatory, antioxidant, and anti-tumorigenic effects [1,15,16].

Cranberry phenolics primarily consist of flavonoids, phenolic acids, and tannins [17]. Among these, flavonoids are the predominant and encompass over 150 identified molecules, grouped into anthocyanins, flavonols, and proanthocyanidins [18]. On overage, Cranberry contains 13 anthocyanins, 16 flavonols, and 26 phenolic acids and benzoates [15]. In various cultivars, 48 polyphenols have been identified, including 19 flavonols, 8 anthocyanins, 7 phenolic acids, and 14 flavan-3-ol oligomers, with relative abundance as follows: flavan-3-ols (41.5–52.2%), flavonols (18.6–30.5%), anthocyanins (8.0–24.4%), and phenolic acids (5.0–12.1%) [19,20].

Regarding oral health, there has been increasing interest in exploring natural bioactive compounds as alternatives or adjuncts to conventional antimicrobials, particularly due to the rise in antibiotic resistance. Cranberry extracts have demonstrated promising effects in this context, encompassing the modulation of host inflammatory response to periodontopathogens, inhibition of biofilm formation and acid production by cariogenic bacteria, suppression of bacterial proteolytic enzymes, and reduction in oxidase stress [21]. These mechanisms are relevant to the control of major oral infectious diseases such as dental caries, periodontal and peri-implant diseases, endodontic infections, and candidiasis [8]. Moreover, recent studies have extended the potential application of Cranberry-derived compounds beyond infection control. Evidence suggests its use in promoting soft and hard tissue healing, reducing inflammation in temporomandibular joint disorders, and even enhancing dentin bond strength following bleaching by neutralizing reactive oxygen species. Such multifunctional properties make Cranberry a promising candidate for integration into preventive, therapeutic, and regenerative dental formulations, including mouth rinses, varnishes, biomaterials, and scaffolds.

Despite this expanding body of research, findings remain fragmented, and the mechanisms underlying Cranberries’ protective effects in the oral cavity are not yet fully elucidated. Therefore, this narrative review aims to systematically analyze and consolidate the available scientific evidence on the utilization of ranberries in the prevention and treatment of oral diseases, highlighting their bioactive constituents, mechanisms of action, and translational potential within dentistry.

## 2. Methods

### 2.1. Search Strategy

A comprehensive literature search was performed in the electronic databases PubMed, Scopus, and Web of Science up to October 2025. The search strategy was based on the MeSH terms and their combinations: “Cranberry” AND “dentistry”. Given the narrative nature of this review, the search strategy was intentionally centered on studies linking Cranberry-derived compounds to dental research, ensuring relevance while avoiding excessively broad searches for a narrative review. Studies were considered eligible if Cranberry and its derived fractions were applied within the context of dentistry, defined as any application involving oral tissues, oral microorganisms, dental materials, oral diseases, or procedures relevant to clinical practice. Eligible study designs included *in vitro*, *in vivo*, and clinical research studies, as long as they reported outcomes directly linked to oral biology or dental interventions. These heterogeneous study designs were included to synthesize evidence, providing a broad overview of the topic and highlighting existing knowledge gaps. Studies were excluded if they were review articles, conference abstracts, book chapters, or if they did not present primary data related to dentistry. Only articles published in English were included, with no restriction regarding the publication date.

### 2.2. Study Selection Process

The selection process was performed in two phases using Rayyan software (Qatar Computing Research Institute, Qatar). In phase 1, two independent reviewers (Y.G.B. and E.B.M.) screened titles and abstracts to identify potentially eligible studies. In phase 2, the same reviewers performed a full-text assessment of selected articles. Any discrepancies between reviewers were resolved through discussion and consensus. These simplified and structured descriptions of the search and selection process were adopted to enhance transparency and to organize the literature within the context of a narrative review.

### 2.3. Data Extraction

From the included studies, the following data were extracted: Author information, publication year, study design, dentistry field, Cranberry properties, study objective, applied tests, and main outcomes.

## 3. Results

### 3.1. Study Selection Outcomes

**Figure 1:**
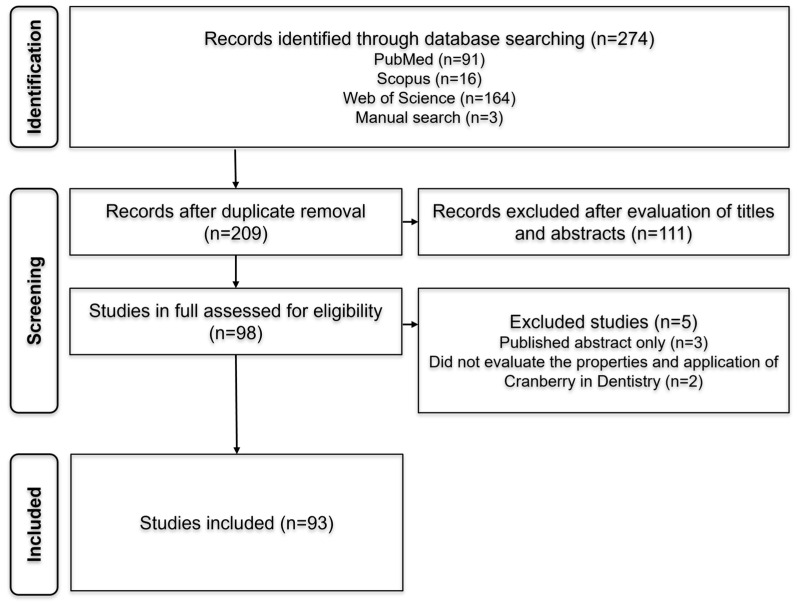
Flowchart of the literature search. References were selected through a two-phase process. Electronic databases (PubMed, Scopus, and Web of Science) were searched up to October 2025.

In phase 1, a total of 274 references were retrieved from the databases: PubMed (91), Scopus (16), and Web of Science (164). Three additional references were identified manually (3). After removing duplicates, 209 articles remained. Following title and abstract screening, 111 studies were excluded based on inter-reviewer agreement, leaving 98 articles for full-text analysis in phase 2. After full-text reading, 5 references were excluded for not meeting the inclusion criteria, resulting in a total of 93 articles eligible for further analysis. A detailed flowchart depicting the identification, inclusion, and exclusion process is presented in Figure 1.

### 3.2. Study Characteristics

**Figure 2:**
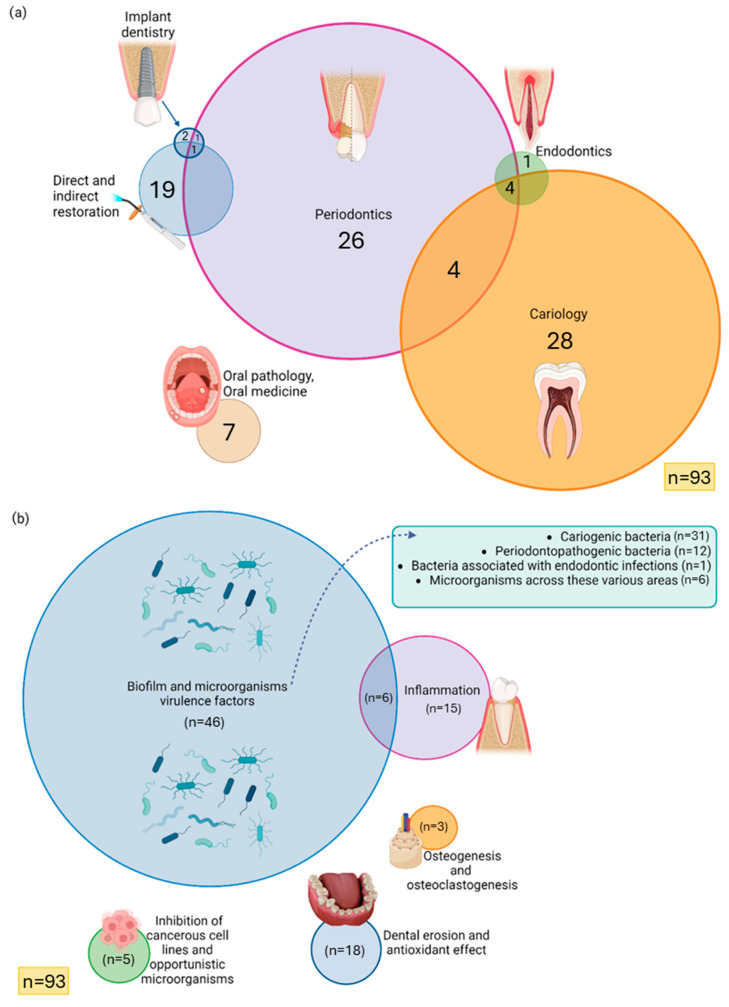
Venn diagram representing the included studies by (**a**) dentistry areas; (**b**) research subject. Each circle represents a specific area or research topic, while overlapping regions indicate studies addressing more than one category simultaneously. The absence of intersection represents a lack of relationship between the respective areas, indicating that no studies met the inclusion criteria for those overlapping categories. Graphical icons (https://www.biorender.com/icon/dental-implant-premolar-in-bone, https://www.biorender.com/icon/dental-curing-light-on, https://www.biorender.com/icon/tooth-premolar-periodontitis-vs-healthy, https://www.biorender.com/icon/apical-periodontitis-primary-incisor, https://www.biorender.com/icon/oral-ulcers, https://www.biorender.com/icon/tooth-generic-molar-cross-section, https://www.biorender.com/icon/microbiome, https://www.biorender.com/icon/tooth-premolar-gingivitis, https://www.biorender.com/icon/osteon, https://www.biorender.com/icon/cranberry?q=cancer-cells-small-tumor) were created with BioRender.com and used under a valid academic license (accessed on 3 November 2025).

The included studies were published from 1998 to 2025. Additionally, as despicable in Figure 2a, the distribution of research fields was as follows: Cariology (n = 28); Periodontics (n = 26); Direct and indirect restoration (n = 19); Oral pathology and Oral medicine (n = 7); Cariology, Periodontics, and Endodontics (n = 4); Periodontics and Cariology (n = 4); Implant dentistry (n = 2); Endodontics (n = 1); Periodontics and Implant dentistry (n = 1); and Periodontics, Implant dentistry, Direct and indirect restoration (n = 1). Most studies evaluated biofilm formation and microbial virulence factors (n = 46), followed by inflammation (n = 15), and inflammation associated with biofilm formation (n = 6), and dental erosion and antioxidant effect (n = 18). Additional topics included cytotoxicity of cancerous cell lines and antifungal effects (n = 5), osteogenesis and osteoclastogenesis (n = 3) (Figure 2b).

**Figure 3:**
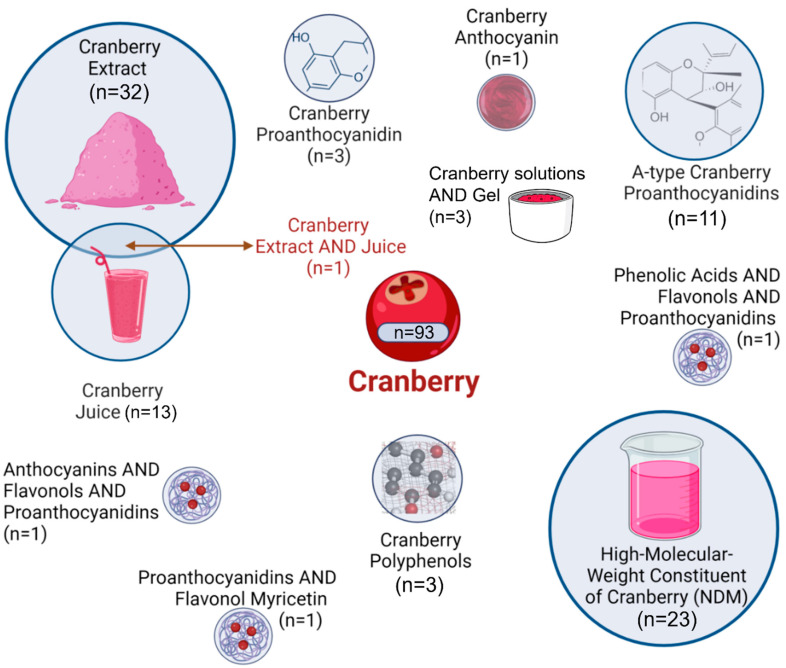
Different Cranberry fractions evaluated in the included studies. Graphical icons (https://www.biorender.com/icon/cranberry, https://www.biorender.com/icon/powder, https://www.biorender.com/icon/smoothie-1, https://www.biorender.com/library#nanoparticle-drug, https://www.biorender.com/icon/beaker-small-full) were created in BioRender.com and used under a valid academic license (accessed on 3 November 2025).

As represented in Figure 3, the most frequently studied Cranberry fractions were Cranberry extract (n = 32) and high-molecular-weight non-dialyzable material (NDM) (n = 23). Other forms included Cranberry juice (n = 13), A-type proanthocyanidins (n = 11), proanthocyanidin fraction (n = 3), polyphenol (n = 3), unspecified Cranberry gels or solutions (n = 3), anthocyanin (n = 1), and several combined formulations. Most investigations were *in vitro* (n = 82), followed by clinical trials (n = 13) and *in vivo* studies (n = 2), with some employing hybrid experimental designs. The main characteristics of the selected studies are summarized in Table 1, Table 2 and Table 3.

### 3.3. Biofilm and Microbial Virulence Factors

Among the 93 studies included, nearly half (n = 46) addressed biofilm formation and microbial virulence. Some of these examined periodontopathogenic bacteria (n = 12) [15,20,23,24,29,30,36,40,43,58,72,77], cariogenic bacteria (n = 31) [22,25,26,27,31,32,39,47,48,57,60,61,62,64,65,70,71,76,92,93,94,95,98], bacteria associated with endodontic infections (n = 1) [67], and a wide range of microorganisms across these various areas (n = 6) [21,39,42,52,66]. Some studies focused on assessing the Cranberry anti-inflammatory properties (n = 6) [40,43,72,76].

**Figure 4:**
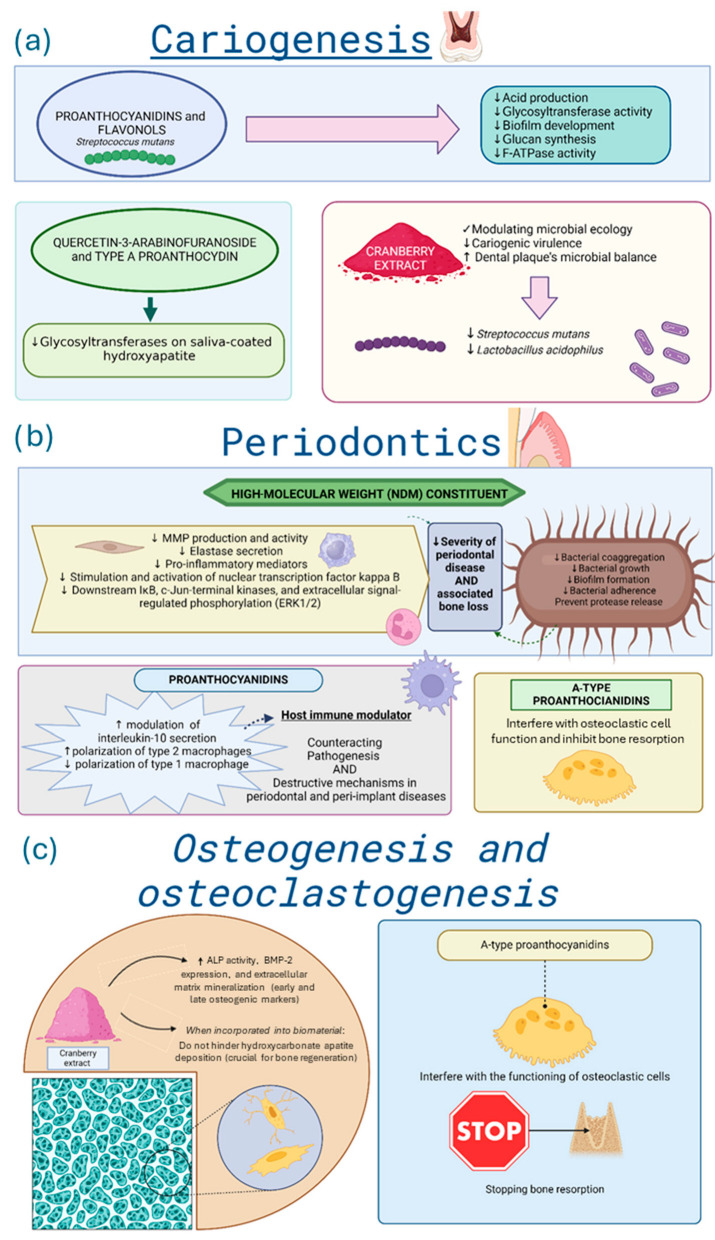
Summary of Cranberry effects on (**a**) cariogenesis, (**b**) periodontics, and (**c**) osteogenesis and osteoclastogenesis. Directional arrows indicate biological modulation, with ↑ representing upregulation or stimulation and ↓ representing downregulation or reduction. Legend: ALP—Alkaline phosphatase; BMP-2—Bone morphogenetic protein 2; MMP—matrix metalloproteinase; F-ATPase—F-Type ATPase. Graphical icons (https://www.biorender.com/icon/tooth-generic-molar-cross-section, https://www.biorender.com/icon/streptococcus, https://www.biorender.com/icon/efb-powder, https://www.biorender.com/icon/bacteria-1-bacillus, https://www.biorender.com/icon/gingival-epithelium, https://www.biorender.com/icon/fibroblast-resting, https://www.biorender.com/icon/macrophage-3d, https://www.biorender.com/icon/neutrophil-editable-837, https://www.biorender.com/icon/macrophage-activated-01, https://www.biorender.com/icon/bacillus-pili, https://www.biorender.com/icon/powder, https://www.biorender.com/library?q=macroporous-scaffold, https://www.biorender.com/icon/osteocyte, https://www.biorender.com/icon/osteoprogenitor-cell, https://www.biorender.com/icon/osteoclast, https://www.biorender.com/icon/stop-sign, https://www.biorender.com/icon/tooth-premolar-bone-woven) were created with BioRender.com and used under a valid academic license (accessed on 3 November 2025).

In cariology, Cranberry-derived proanthocyanidins and flavonols exhibited strong *in vitro* inhibitory effects on *Streptococcus mutans* virulence, including reduced acid production, glycosyltransferase activity, and biofilm development on tooth surfaces [4,22,32,48], as observed in Figure 4a. These beneficial effects seem to be related to inhibition of glucan synthesis, F-ATPase activity, and acid production [4,26,32,48,105]. Specific fractions from Cranberry extracts, such as quercetin-3-arabinofuranoside and A-type proanthocyanidin, exhibited potent *in vitro* inhibition of glycosyltransferases on saliva-coated hydroxyapatite [52,93,106]. These bioactive fractions also interfered with bacterial adhesion and reduced the acidogenic potential of dental biofilms in controlled environments [3,48,61].

Cranberry extracts inhibited *Lactobacillus acidophilus*, indicating broader modulation of the oral microbiota *in vitro* [64]. Clinically, Cranberry-based mouthwashes decreased *Streptococcus mutans* and total bacteria counts in saliva, showing comparable efficacy to 0.2% chlorhexidine [95]. Pediatric formulations achieved similar benefits without adverse effects in clinical [94] and laboratory conditions [70]. When incorporated into dentifrices, Cranberry extracts clinically modulated plaque ecology and reduced cariogenic virulence [98]. Overall, these findings support Cranberries as a natural, non-toxic, and effective adjunct for preventing dental caries.

In periodontitis, as described in Figure 4b, high-molecular-weight Cranberry fractions (NDM) inhibited *in vitro* bacterial coaggregation and adhesion, protease release by major periodontal pathogens [23,28,29]. Clinically, Cranberry mouthwash reduced plaque and gingival indices comparably to chlorhexidine [95,107]. *In vitro*, A-type proanthocyanidins also disrupted *Candida albicans* biofilms [43], likely mediated by Cranberries’ capacity to inhibit the activation of nuclear factor B p65, which influences the virulence of *Candida albicans* and attenuates inflammation. Cranberry proanthocyanidins and flavonoids incorporated into self-curing polymethylmethacrylate resin used in prostheses reduced the number of colony-forming units of *Candida albicans* in a laboratory environment [5,91].

### 3.4. Osteogenesis and Osteoclastogenesis

Only three studies addressed bone-related mechanisms *in vitro* [16,54,83]. One reported that incorporating Cranberry extract into mesoporous bioactive glass preserved hydroxycarbonate apatite deposition, maintaining material bioactivity [16]. The second demonstrated that A-type proanthocyanidins inhibited osteoclastic activity, suggesting a potential role in preventing bone resorption associated with periodontitis [54]. Also, the Cranberry extract increased early and late osteogenic markers, such as alkaline phosphatase and extracellular matrix mineralization *in vitro* [83]. For more details, see Figure 4c.

### 3.5. Inflammation

Several articles (n = 21) investigated the Cranberries’ anti-inflammatory effects [28,40,43,72,76,99]. Six overlapped with biofilm-focused research [40,43,72,76,100,102].

Clinically, five studies investigated Cranberries in the context of gingival inflammation. Overall, the evidence suggests potential benefits, although with important methodological limitations across studies. A nature-based gel achieved clinical outcomes comparable to conventional dentifrices while indicating a possible host-response–modulating effect [102]. A multinutrient supplement containing Cranberry extract improved periodontal clinical parameters, albeit without statistically significant differences compared with placebo [100]. Other trials reported reductions in plaque accumulation and gingivitis scores in patients undergoing fixed orthodontic treatment [50], and omega-3–enriched Cranberry juice was associated with reduced glycated hemoglobin, increased HDL-C levels, and improved periodontal conditions [49]. Additionally, an eight-week intake of a Cranberry-based functional beverage reduced gingival inflammation, plaque index, and approximal plaque index; however, this comparison was made against a water control, which is not an adequately matched comparator and limits the interpretability of the findings [37].

Laboratory-based studies demonstrated that NDM displayed protective effects on macrophages stimulated by lipopolysaccharides (LPS) from periodontopathogens [28], inhibited matrix metalloproteinase (MMP) production and activity in LPS-stimulated fibroblasts and macrophages [33,34,50], suppressed neutrophil elastase [19], and inhibited the secretion of pro-inflammatory mediators (interleukin-6, interleukin-8, and prostaglandin E2) by gingival fibroblasts [45,46] and epithelial cells [63]. NDM also inhibited the activation of nuclear transcription factor kappa B [51], IκB, and MAPK pathways, including c-Jun-terminal kinases and extracellular signal-regulated phosphorylation kinases 1 and 2 (ERK1/2) in Smulow-Glickman cells [73]. Proanthocyanidins stimulated interleukin-10 secretion, induced M2 macrophage polarization, and suppressed M1 macrophage polarization [53]. Collectively, these laboratory findings suggest that Cranberry compounds can act as a host immune modulator in periodontal and peri-implant inflammation.

In addition, NDM mitigated *in vitro* temporal mandibular joint inflammation, downregulating interleukin 1β, IL-6, IL-8, vascular endothelial growth factor (VEGF), and transcription factors such as kappa B nuclear transcription factor and activator protein 1. As a result, NDM holds potential therapeutic use in inflammatory arthropathies of the temporomandibular joint [75].

Although two studies reported an increase in IL-6 after the deacidification of Cranberry juice [59,74], which is an effect inconsistent with anti-inflammatory activity, this response seems to reflect alterations in the chemical composition of the juice rather than the inherent properties of Cranberry-derived compounds. Most other studies using native extracts or purified fractions demonstrated reductions in IL-6, IL-8, VEGF, and related mediators, suggesting that these effects may be model- and preparation-dependent. Given that the evidence is predominantly derived from laboratory experiments, these findings should not be extrapolated to clinical contexts, even though they collectively suggest a potential anti-inflammatory influence under specific experimental conditions.

### 3.6. Dental Erosion and Antioxidant Effect

**Figure 5:**
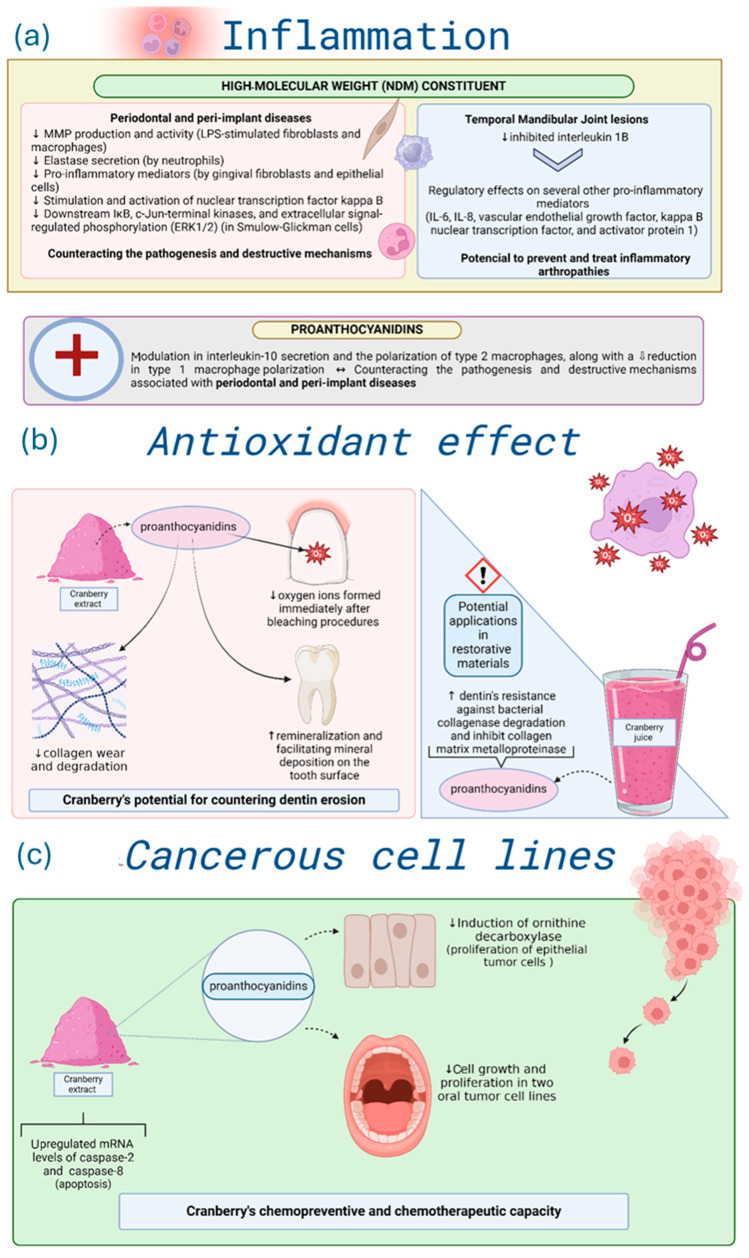
Summary of Cranberry effects on (**a**) inflammation, (**b**) antioxidant effect, and (**c**) cancerous cell lines. Directional arrows indicate biological modulation, with ↑ representing upregulation or stimulation and ↓ representing downregulation or reduction. Graphical icons (https://www.biorender.com/library?q=immune-cells-inflammation, https://www.biorender.com/icon/fibroblast-resting, https://www.biorender.com/icon/macrophage-3d, https://www.biorender.com/icon/neutrophil-editable-837, https://www.biorender.com/icon/collagen-matrix?q=extracellular-matrix-collagen-network, https://www.biorender.com/icon/tooth-incisor-healthy, https://www.biorender.com/icon/tooth-molar, https://www.biorender.com/sub-categories/cytoskeleton-and-ecm?q=oxidative-stress, https://www.biorender.com/icon/smoothie-1, https://www.biorender.com/icon/powder, https://www.biorender.com/icon/pseudostratified-epithelium-few-cells-not-ciliated, https://www.biorender.com/icon/mouth-open-01, https://www.biorender.com/sub-categories/cytoskeleton-and-ecm?q=cancer-cells-metastisizing) were created with BioRender.com and used under a valid academic license (accessed on 3 November 2025).

Cranberry’s role as an antioxidant and in dental erosion prevention was also evaluated (n = 18), with all studies showing *in vitro* results [7,89,90,96,97,101,103]. As depicted in Figure 5b, Cranberry inhibited dentin erosion [7,56,69] but had limited effects on enamel erosion [22,41]. *In vitro* findings indicate that Cranberries neutralized residual oxygen radicals following dental bleaching, enhancing adhesive bond strength [55,108]. This is attributed to oligomeric proanthocyanidins, which donate electrons to the bleached surface and promote radical scavenging through epicatechin–gallic acid esterification [55,108]. These proanthocyanidins also supported dentin remineralization and inhibited collagen degradation, thus reducing structural wear [7,25,41]. Nevertheless, the low pH of Cranberry extracts may counteract their protective effects on enamel [22]. Overall, Cranberries’ antioxidant capacity and MMP inhibition make them a promising additive for adhesives and restorative materials [69].

### 3.7. Inhibition of Cancerous Cell Lines

Cranberry potential in controlling cancerous cell lines was addressed *in vitro* (n = 5) [6,26,27]. The primary objective, as highlighted in three of the articles, was to assess the chemopreventive and chemotherapeutic potential of Cranberry polyphenols at various stages of oral carcinogenesis. Notably, it was reported that proanthocyanidins effectively inhibit the induction of ornithine decarboxylase, an enzyme involved in the proliferation of epithelial tumor cells [28]. *In vitro* evidence suggests that administering proanthocyanidins derived from Cranberry extract significantly reduces cell growth and proliferation in two oral tumor cell lines. Furthermore, within just 24 h, the Cranberry extract upregulated the mRNA levels of caspase-2, an initiator of apoptosis, and caspase-8, an effector. Moreover, the extract demonstrated the ability to decrease cell adhesion in both cell lines. These findings highlight Cranberry extract’s chemopreventive and chemotherapeutic capacity, selectively targeting cancer cells without affecting normal cells and tissues [26]. Another *in vitro* study reinforces these findings by demonstrating the effect of Cranberry extract in reducing tumor cell viability and proliferation while maintaining the viability of normal cells [6]. For more details, see Figure 5c.

## 4. Overview on the Use of Cranberries in Dentistry

Collectively, the reviewed evidence demonstrates that Cranberry possesses multifaceted therapeutic properties, including antimicrobial, anti-inflammatory, and antioxidant properties. Among various Cranberry fraction types, the Cranberry extract (n = 32) and high-molecular-weight non-dialyzable material (NDM) were the most studied (n = 23). These components were found to exert significant effects, particularly on oral pathogens such as *Streptococcus mutans* and periodontopathogenic bacteria. Other fractions, including proanthocyanidins and flavonols, have also shown promise in inhibiting biofilm formation and bacterial coaggregation, crucial factors in dental caries and periodontitis development.

Additionally, proanthocyanidins and flavonols demonstrated specific efficacy against *Streptococcus mutans*, a key player in dental caries, by inhibiting acid production and biofilm development on tooth surfaces. Cranberry fractions presented inhibitory effects on glycosyltransferase activity, essential for glucan synthesis, and on F-ATPase activity, reducing acidogenicity and cariogenic potential. Concerning periodontopathogenic bacteria, the high-molecular-weight constituents of Cranberries effectively inhibited biofilm formation and bacterial adherence related to periodontal disease. Studies revealed that Cranberry extracts reduced plaque index and gingival inflammation in clinical trials, suggesting their potential as a therapeutic agent in periodontal disease management. Cranberry extracts, particularly A-type proanthocyanidins, showed potential in promoting bone regeneration and inhibiting osteoclast activity. These findings suggest applications in treating periodontitis and promoting osseointegration in dental implants. Cranberry extract also demonstrated antifungal properties against *Candida albicans*, with potential applications in prosthetic dentistry. Incorporating Cranberry-derived proanthocyanidins into acrylic resins used in dental prostheses effectively reduced *Candida* colonization.

The anti-inflammatory effects of Cranberries were linked to their ability to suppress matrix metalloproteinase activity and inhibit the production of pro-inflammatory mediators. Moreover, its antioxidant properties were beneficial in preventing dentin erosion and enhancing the bond strength of dental materials after bleaching. Studies also revealed that Cranberry polyphenols, particularly proanthocyanidins, exhibited chemopreventive and chemotherapeutic effects by inhibiting tumor cell proliferation and inducing apoptosis in oral cancer cell lines. These properties underscore Cranberry’s potential as a complementary therapeutic agent in oral cancer management.

One limitation of this review is that most studies included are based on *in vitro* analyses (n = 82), with a limited number of clinical trials available (n = 13, including 2 hybrid studies combining *in vitro* and clinical designs). While these laboratory studies are valuable for understanding the potential effects of Cranberries in a controlled environment, they do not fully reflect their efficacy in real-world clinical settings. Therefore, further clinical trials are essential to validate these findings and determine the true therapeutic potential of this specific berry in dentistry. Consequently, the results presented in this review should be interpreted with caution, as the predominance of *in vitro* evidence limits the extent to which clinical applicability can be inferred at this stage.

## 5. Perspectives on Cranberry Applications in Dentistry

Despite encouraging *in vitro* results, several formulation challenges must be resolved before Cranberry extracts can be incorporated into dental products. Polyphenols are chemically unstable and highly sensitive to pH, temperature, oxygen, and light, which may reduce their bioactivity in commercial formulations. Compatibility issues with common excipients in dentifrices, gels, and mouthwashes (including surfactants, thickeners, and preservatives) may further affect their stability or performance. Additionally, the natural variability of Cranberry extracts, driven by fruit origin, processing methods, and differing concentrations of active compounds, complicates standardization and reproducibility.

Safety considerations are also critical, as the antimicrobial and antibiofilm activity of Cranberry extracts is dose-dependent, with stronger effects observed at higher concentrations of their active polyphenols. However, long-term safety data for topical oral applications remain scarce, and no reference thresholds have been established for dental formulations. This dose dependence, combined with the substantial variability in extraction and concentration protocols, limits the comparability of published studies and challenges the translation of effective *in vitro* concentrations into clinically acceptable products.

Given these limitations, future research should prioritize clinical validation of Cranberry-derived mouthwashes, dentifrices, and restorative materials. The incorporation of proanthocyanidins and NDM into oral care formulations could offer a natural, biocompatible alternative to conventional antimicrobials such as chlorhexidine. Moreover, the antioxidant and bond-strength-enhancing effects of Cranberry compounds could be leveraged to develop advanced adhesive and restorative systems with greater longevity. Overall, Cranberries hold great promise for advancing preventive and therapeutic strategies in dentistry, emphasizing a bio-based, non-toxic approach to oral health care.

## 6. Conclusions

Growing evidence suggests that Cranberry bioactives, particularly flavonols, anthocyanidins, and proanthocyanidins, may exert promising biological effects relevant to dentistry, including antimicrobial, anti-inflammatory, antioxidant, and antiproliferative actions in preclinical models. These activities have been demonstrated predominantly *in vitro*, with limited findings in animal or clinical research. Therefore, while these compounds show potential as multifunctional agents, their clinical applicability remains uncertain.

Given their versatility, further translational, mechanistic, and product-development research is warranted to determine whether Cranberry-derived agents can be integrated into both clinical practice and daily oral care in the future.

## Figures and Tables

**Table 1:** Summary of *in vivo* (hybrid *in vivo* and *in vitro*) included studies’ descriptive characteristics in chronological order.

Author (Year) Cranberry Fraction (*Vaccinium macrocarpon* Specie)	Type of Study	Aim	Outcomes	Reference
Koo, H. et al. (2010) *Proanthocyanidins*	*In vitro* *In vivo*	To investigate the effect of a highly purified and chemically characterized Cranberry proanthocyanidin fraction on *Streptococcus mutans* biofilm formation on saliva-coated hydroxyapatite surface, and on dental caries development process in Sprague-Dawley rats.	Cranberry proanthocyanidins reduced *Streptococcus mutans* biofilm formation *in vitro* and dental caries development *in vivo*, effects that may be attributed to specific bioactive A-type dimers and oligomers.	[22]
Polak, D. et al. (2013) NDM	*In vitro* *In vivo*	To evaluate the NDM effect on the virulence of a mixed infection caused by *Porphyromonas gingivalis* and *Fusobacterium nucleatum* in mice.	NDM inhibited the adhesion of both bacterial species to epithelial cells and reduced their coaggregation in a dose-dependent manner. It also eliminated TNF-α expression in macrophages exposed to *Porphyromonas gingivalis* and *Fusobacterium nucleatum*, without affecting cell viability. In mice, NDM intake attenuated periodontitis severity and lowered TNF-α levels.	[20]

**Legend:** NDM—high-molecular-weight non-dialyzable material; TNF-α—Tumor necrosis factor-alpha.

**Table 2:** Summary of included *in vitro* studies’ descriptive characteristics in chronological order.

Author (Year) Cranberry Fraction (*Vaccinium macrocarpon* Specie)	Type of Study	Aim	Outcomes	Reference
Weiss, E.I. et al. (1998) NDM	*In vitro*	To evaluate the capacity of the NDM derived from Cranberry juice to hinder the coaggregation of specific oral bacterial strains.	NDM reversed the coaggregation of 49 bacterial pairs, of a total of 84 tested.	[23]
Yamanaka, A (2004) NDM	*In vitro*	To assess the capacity of Cranberry juice to inhibit the adhesion of [^3^H]-thymidine-labeled oral *Streptococcus* strains to saliva-coated hydroxyapatite beads.	NDM inhibited biofilm formation by oral *Streptococci*, including cariogenic strains, indicating that daily consumption of Cranberry juice may reduce dental plaque development.	[24]
Steinberg, D. et al., (2004) NDM	*In vitro*	To evaluate the impact of the NDM on dental biofilm components, including the activities of glucosyltransferase (GTF), fructosyltransferase (FTF), as well as the adhesion capacity of *Streptococcus sobrinus*.	NDM inhibited the activity of both immobilized and soluble GTF and FTF enzymes. Additionally, NDM reduced the adhesion of *Streptococcus sobrinus* to hydroxyapatite.	[25]
Koo, H. et al. (2005) Cranberry juice	*In vitro*	To assess the effect of Cranberry juice on the activity of specific GTF enzymes (GTF B, C, and D) adsorbed onto hydroxyapatite surfaces, and to determine the impact of short-term daily exposure to Cranberry on the development, glucan composition, and acidogenicity of *Streptococcus mutans*.	Cranberry juice inhibited glucan-mediated biofilm formation and acid production, demonstrating potential as a natural agent for preventing biofilm-related oral diseases. Additionally, Cranberry blocked bacterial adherence mediated by surface glucans and reduced *Streptococcus mutans* biofilm formation, acidogenicity, and insoluble glucan content.	[26]
Steinberg, D. et al. (2005) NDM	*In vitro*	To assess the anti-adhesion activity of the NDM against *Streptococcus sobrinus*.	NDM promoted desorption of *Streptococcus sobrinus* both in the presence and absence of extracellular glucans and frutans. Pre-coating the bacteria with NDM reduced their biofilm-forming capacity.	[27]
Bodet, C. et al. (2006) NDM	*In vitro*	To evaluate the influence of NDM on the macrophage proinflammatory cytokine response triggered by lipopolysaccharides (LPS) derived from periodontopathogenic bacteria.	NDM was shown to be a potent inhibitor of the pro-inflammatory cytokine and chemokine responses induced by LPS.	[28]
Labrecque, J. et al. (2006) NDM	*In vitro*	To investigate the NDM effect on biofilm development, growth, and adhesion characteristics of *Porphyromonas gingivalis*.	NDM was shown to be a potent inhibitor of biofilm formation by *Porphyromonas gingivalis* and reduced *Porphyromonas gingivalis* attachment to surfaces coated with type I collagen, fibrinogen, or human serum.	[29]
Bodet, C. et al. (2006) NDM	*In vitro*	To study the NDM impact on the proteolytic activity of periodontopathogenic bacteria.	NDM reduced the proliferation of *Porphyromonas gingivalis*, *Treponema forsythia*, and *Treponema denticola* in periodontal pockets, as well as their proteinase-mediated destructive activity associated with periodontitis.	[30]
Duarte, S. et al. (2006) Anthocyanins, flavonols, proanthocyanidins	*In vitro*	To assess the effects of flavonol, anthocyanin, and proanthocyanidin extracts on virulence factors associated with biofilm formation and acid production of *Streptococcus mutans*.	Flavonols and proanthocyanidins, individually or combined, inhibited the activities of surface-adsorbed glucosyltransferases and F-ATPases, as well as acid production by *Streptococcus mutans*.	[31]
Gregoire, S. et al. (2007) Phenolic acids, flavonols, proanthocyanidins	*In vitro*	To evaluate the effect of phenolic compounds on the virulence traits of *Streptococcus mutans* related to glucan production and acidogenic potential.	Specific flavonoids demonstrated biological activity against *Streptococcus mutans*. The activity of Cranberry extracts may be attributed to the complex mixture of flavonoids rather than a single active compound.	[32]
Bodet, C. et al. (2007) NDM	*In vitro*	To evaluate the influence of the NDM on the enzymatic activities of MMP-3, MMP-9, and elastase, and on the synthesis of MMP by human cells stimulated with LPS from *Aggregatibacter actinomycetemcomitans*.	NDM demonstrated strong inhibitory effects on MMP-3, MMP-9, and elastase production and activity in macrophages and gingival fibroblasts.	[33]
Bodet, C. et al. (2007) NDM	*In vitro*	To assess NDM influence on the inflammatory mediators production (IL-6, IL-8, and PGE2) by human gingival fibroblasts stimulated with LPS from *Aggregatibacter actinomycetemcomitans*.	NDM inhibited IL-6, IL-8, and PGE2 production by gingival fibroblasts, suggesting that NDM may exert a beneficial effect in slowing periodontal disease progression when used alongside conventional therapy.	[34]
Yamanaka, A. et al. (2007) Cranberry polyphenol fraction	*In vitro*	To determine the effect of the polyphenolic fraction of Cranberries on cell surface hydrophobicity, biofilm formation, and growth of *Streptococcus mutans* strains.	Cranberry polyphenolic fraction reduced the hydrophobicity of *Streptococcus sobrinus* and *Streptococcus mutans* in a dose-dependent manner and inhibited biofilm formation. Additionally, the growth of *Streptococcus mutans* was suppressed.	[35]
La, V.D. et al. (2009) Proanthocyanidin-rich fraction	*In vitro*	To investigate the protective capacity of Cranberries in preventing cytotoxic effects induced by bacterial components on monocyte-derived macrophages and oral epithelial cells.	Cranberry polyphenols demonstrated a protective capacity against toxicity induced by bacterial components on host cells.	[36]
La, V.D. et al. (2009) A-type proanthocyanidins	*In vitro*	To assess AC-PACs’ effects on the production of different MMPs by human monocyte-derived macrophages stimulated with LPS from *Aggregatibacter actinomycetemcomitans*, and on the catalytic activity of recombinant MMP-1 and MMP-9.	Cranberry suppressed MMP production and activity, indicating potential for developing host-modulating strategies to prevent MMP-mediated tissue destruction in periodontitis.	[37]
Feldman, M. et al. (2009) NDM	*In vitro*	To analyze the potential anti-adhesive action of the NDM by examining its effect on secretion, gene expression, and promoter activity of the fructosyltransferase.	NDM reduced the secretion of extracellular fructosyltransferase in a dose-dependent manner. It also significantly decreased luciferase activity under the control of the fructosyltransferase promoter.	[38]
Feldman, M. et al. (2010) NDM	*In vitro*	To evaluate the capacity of the NDM to bind to immobilized fructosyltransferase and inhibit its enzymatic activity.	One anti-biofilm mechanism of NDM involved an immediate and irreversible inhibition of immobilized fructosyltransferase activity, attributed to its strong binding affinity for the enzyme.	[39]
La, V.D. et al. (2010) A-type proanthocyanidins	*In vitro*	To assess the Cranberry effects on key virulence factors of *Porphyromonas gingivalis* and on the inflammatory response elicited in oral epithelial cells upon stimulation by this bacterium.	Cranberry diminishes the virulence of *Porphyromonas gingivalis* by inhibiting biofilm formation, adhesion, proteinase activity, and invasiveness. Additionally, Cranberry exhibited anti-inflammatory effects by suppressing the *Porphyromonas gingivalis*-induced inflammatory response in human oral epithelial cells.	[40]
Tanabe, S. et al. (2011) A-type proanthocyanidins	*In vitro*	To study the impact of AC-PACs on osteoclast differentiation and bone resorption activity.	AC-PACs interfered with osteoclastic cell maturation and physiology, and prevented bone resorption.	[16]
Chatelain, K. et al. (2011) Cranberry extract	*In vitro*	To analyze the effects of Cranberry and grape seed extracts on the proliferation of oral squamous cell carcinoma cells.	Cranberry extracts inhibited the proliferation of CAL27 cells and SCC25 cells.	[41]
Babu, J. et al. (2012) NDM	*In vitro*	To assess the effect of low concentrations of the NDM on the metabolic activity and biofilm formation of *Streptococcus gordonii* on restorative surfaces.	NDM selectively inhibited the metabolic activity of *Streptococcus gordonii*, without affecting bacterial viability on dental surfaces (including titanium implant, amalgam, and composite materials).	[42]
Feldman, M. et al. (2012) A-type proanthocyanidins	*In vitro*	To evaluate the potential synergistic effects of AC-PACs and licochalcone A against *Porphyromonas gingivalis* and the associated inflammatory response in a macrophage model.	AC-PACs did not affect the growth of *Porphyromonas gingivalis*, but reduced bacterial adherence. Additionally, AC-PACs and licochalcone A demonstrated synergistic anti-bacterial, anti-adherence, and anti-inflammatory activities.	[43]
Feldman, M. et al. (2012) A-type proanthocyanidins	*In vitro*	To understand the influence of AC-PACs on the pathogenic traits of *Candida albicans* and on the inflammatory response of oral epithelial cells.	AC-PACs inhibited *Candida albicans* adherence to oral epithelial cells and reduced IL-6 and IL-8 secretion in a dose-dependent manner during infection of epithelial cells with *Candida albicans*.	[44]
Tipton, D. et al. (2013) NDM	*In vitro*	To assess the Cranberry effects on the production of IL-6, IL-8, and IL-17 by human gingival epithelial cells and fibroblasts.	NDM inhibited the secretion of IL-17, IL-6, and IL-8 by gingival fibroblasts and epithelial cells.	[19]
Tipton, D. et al. (2013) NDM	*In vitro*	To evaluate the cytotoxicity of Cranberry and of LPS from the periodontopathogens *Fusobacterium nucleatum* and *Porphyromonas gingivalis* in fibroblasts, as well as to assess the effects of Cranberry components on fibroblast viability, NF-κB activation, and the production of IL-6 and MMP-3 following LPS stimulation.	Low concentrations of NDM did not exhibit toxicity and inhibited NF-κB and MMP-3, suggesting that Cranberry components modulated fibroblast inflammatory responses.	[45]
Tipton, D. et al. (2014) NDM	*In vitro*	To assess the effects of Cranberries on NF-κB and MAPK/AP-1 pathway activation involved in IL-6 production by gingival epithelial cells stimulated with IL-1β.	NDM decreased nuclear levels of IL-1β-activated NF-κB (p65) and AP-1 (phospho-c-Jun) and strongly inhibited IL-6 production. The absence of effects on IκBα, c-Jun, or ERK1/2 phosphorylation suggested that NDM acted downstream in these pathways, possibly via ubiquitination and proteosomal degradation of IκBα or inhibition of nuclear activity of c-Jun and/or ERK1/2 in S-G cells.	[46]
Hrynash, H. et al. (2014) Anthocyanin-rich extract incorporated in a resin	*In vitro*	To determine bacterial growth inhibition, mechanical characteristics, and the release rate and stability of compounds in copolymers incorporated with anthocyanins.	The anthocyanin-rich extract inhibited *Streptococcus mutans* growth to a lesser extent than chlorhexidine.	[47]
Kim, D. et al. (2015) Proanthocyanidins tetramer (DP4) and monomer (DP9), and flavanol myricetin	*In vitro*	To evaluate the effect of flavonoids on disrupting the accumulation and survival of *Streptococcus mutans* and on the mechanical stability of the biofilm-apatite interface.	Cranberry disrupted biofilm architecture, resulting in a defective matrix and failure to form microcolonies on the sHA surface. Topical applications of Cranberry flavonoids weakened biofilm mechanical stability and increased pH at the biofilm-apatite interface compared to vehicle-treated biofilms (vehicle comprised 5 mM phosphate-buffered solution containing 20% EtOH and 0.8% DMSO).	[48]
Lombardo, B. et al. (2015) A-type proanthocyanidins	*In vitro*	To assess the potential synergistic action of AC-PACs and EGCG with LL-37 in reducing the secretion of inflammatory mediators by oral mucosal cells.	AC-PACs reduced the secretion of G-CFS, GRO-α, IL-8, IP-10, and MCP-1, while not affecting the IL-6 and MMP secretion. The combination of AC-PACs or EGCG and LL-37 synergistically inhibited inflammatory cytokine release.	[49]
Tipton, D. et al. (2016) NDM	*In vitro*	To examine the effects of Cranberry on IL-6 and MMP-3 production by gingival fibroblasts exposed to representative AGEs formed during diabetic hyperglycemia, specifically G-HSA, or to LPS with or without G-HSA.	NDM inhibited IL-6 and MMP-3 production in the presence of either G-HSA or LPS, suggesting that Cranberry phenols may have modulated the host response and could be beneficial in managing periodontitis in patients with poorly controlled diabetes.	[50]
Tipton, D. et al. (2016) NDM	*In vitro*	To evaluate the NDM effects on IL-6, IL-8, and VEGF production by human TMJ synovial fibroblast-like cells stimulated by IL-1β.	NDM did not affect fibroblast viability, while it inhibited IL-1β-stimulated IL-6, IL-8, and VEGF production. NDM also reduced nuclear levels of NF-kB and AP-1. These findings suggested that NDM may serve as a host-modulatory agent for preventing or treating inflammatory arthropathies of the TMJ.	[51]
Neto, C. et al. (2017) NDM	*In vitro*	To identify the polyphenolic and non-polyphenolic constituents present in the NDM.	The crude NDM and NDMac demonstrated equal effectiveness in inhibiting coaggregation of *Fusobacterium nucleatum* with *Streptococcus sanguinis* or *Porphyromonas gingivalis*. NDMac also reduced biofilm formation. The anti-adhesion effects of NDM on oral bacteria appeared to result from a combination of polyphenol and non-polyphenol constituents.	[52]
Rajeshwari, H.R. et al. (2017) Cranberry juice concentrate, and thermoreversible gel of Cranberry juice concentrate	*In vitro*	To determine the efficacy of a thermoreversible gel formulated with Cranberry juice concentrate as a local drug delivery system for periodontitis treatment.	Cranberry exhibited inhibitory effects against *Porphyromonas gingivalis* virulence factors. Cranberry juice concentrate and 0.2% chlorhexidine gluconate gel showed similar inhibition against *Streptococcus mutans*, *Enterococcus faecalis*, *Aggregatibacter actinomycetemcomitans*, *Porphyromonas gingivalis*, and *Tannerella forsythia*.	[15]
Boteon, A.P. et al. (2017) 10% Cranberry extract gel	*In vitro*	To assess the effects of gels enriched with Cranberry and grape seed extracts on the inhibition of dental wear and degradation of the demineralized organic matrix.	Cranberry extract and sodium fluoride demonstrated comparable reductions in dentin wear and degradation of demineralized organic matrix. These results suggested that Cranberries could serve as a promising natural agent for the prevention of dentin erosion.	[53]
Galarraga-Vinueza, M.E. et al. (2018) A-type proanthocyanidins	*In vitro*	To evaluate the potential of 58S mesoporous bioactive glass particles incorporating Cranberry and propolis to generate antibiofilm compounds.	Bioactive glass incorporating Cranberry and propolis showed an increase in hydroxyapatite crystal formation.	[54]
Khairnar, M. et al. (2018) Cranberry extract	*In vitro*	To analyze the anti-cancer activity of Cranberry and chlorhexidine against oral cancer cell lines.	Cranberry did not exhibit anti-tumoral effect against the AW13516 cell line, whereas it demonstrated growth inhibition of the KB cell line.	[55]
Nandakumar, M.; Nasim, I. (2018) Cranberry extract	*In vitro*	To examine the effectiveness of grape seed and Cranberry extracts in preventing dental erosion using optical emission spectrometry.	The protective activities of grape seed extract and Cranberry extract were inferior to those of the stannous fluoride control group in preventing enamel erosion.	[56]
Abu-obaid, E. et al. (2019) Cranberry extract	*In vitro*	To evaluate the antimicrobial activity of various natural and semi-natural mouthrinses against *Streptococcus mutans* isolated from the saliva of Saudi children, as well as against reference *Streptococcus mutans* strains.	Mouthrinses formulated with an herbal mix and Cranberry demonstrated potential as effective natural alternatives to chlorhexidine mouthrinses.	[57]
Ben Lagha, A. et al. (2019) Proanthocyanidins	*In vitro*	To assess the Cranberry proanthocyanidins’ impacts on the gene expression and cytotoxic activity of LtxA.	Cranberry proanthocyanidins reduced gene expression in *Aggregatibacter actinomycetemcomitans* and neutralized the cytolytic and pro-inflammatory responses of human macrophages exposed to LtxA.	[58]
Eggula, A. et al. (2019) Cranberry extract	*In vitro*	To investigate the neutralizing effect of a 6% Cranberry solution on the bond strength of bleached enamel in comparison with a 10% sodium ascorbate solution.	Sodium ascorbate solution demonstrated the greatest ability to restore compromised bond strength following bleaching treatment.	[59]
Kumar, V. et al. (2019) Cranberry extract	*In vitro*	To determine the *in vitro* antimicrobial efficacy of hydro-alcoholic Cranberry extract against Socransky complexes and the predominant cariogenic, mycotic, and endodontic microbial communities of the oral cavity.	Cranberry extract showed satisfactory inhibitory and bactericidal effects against all test pathogens compared with the negative control, which contained only the bacterial suspension.	[21]
Philip. N. et al. (2019) Extracts of Cranberry, blueberry, and strawberry	*In vitro*	To assess the potential inhibition of *Streptococcus mutans* biofilm by Cranberry, blueberry, and strawberry extracts separately, and in combination (Orophenol^®^).	Cranberry and Orophenol^®^ extracts affected bacterial metabolic activity, acid production, and bacterial/exopolysaccharide biovolumes. Cranberry extract proved most effective in disrupting the virulence factors of *Streptococcus mutans*, without affecting bacterial viability.	[60]
Philip. N. et al. (2019) A-type proanthocyanidins	*In vitro*	To study the impact of Cranberry extracts on saliva-derived polymicrobial biofilms with respect to biofilm biomass, acidogenicity, exopolysaccharide and microbial biovolumes, colony-forming units, and the proportion of bacterial species related to caries and oral health.	Cranberry extract reduced biofilm biomass, acidogenicity, exopolysaccharide and microbial biovolumes, and colony-forming units, while inducing beneficial ecological shifts in saliva-derived polymicrobial biofilms.	[61]
Philip. N. et al. (2019) Cranberry extract	*In vitro*	To assess the effects of Cranberry extracts on dual-species *Streptococcus mutans-Candida albicans* biofilms associated with the severity of early caries during childhood.	Cranberry extracts demonstrated inhibition of cariogenic virulence factors in *Streptococcus mutans-Candida albicans* biofilm.	[4]
Kokubu. E. et al. (2019) Cranberry extract	*In vitro*	To evaluate and compare the effects of polyphenol-rich lingonberry extract and Cranberry juice on oral *Streptococcus* species.	Cranberry and lingonberry fractions at 0.5–1 mg/mL significantly reduced biofilm formation by *Streptococcus mutans*, *Streptococcus sobrinus*, and *Streptococcus sanguinis*. Conversely, fractions at 0.5–2 mg/mL increased biofilm formation by *Streptococcus mutans* and *Streptococcus sobrinus*, but not by *Streptococcus sanguinis*. Fractions at 1–2 mg/mL diminished the bioactivity of *Streptococcus mutans*, whereas the 0.5 mg/mL fraction enhanced the bioactivity of all tested strains.	[62]
Philip. N. et al. (2020) Cranberry extract	*In vitro*	To investigate whether experimental natural products may selectively inhibit the growth of *Streptococcus mutans* without compromising the viability of the health-associated oral commensal *Streptococcus sanguinis*.	The tested fruit berry extracts (Cranberry, blueberry, and strawberry) failed to inhibit the growth of *Streptococcus mutans* and *Streptococcus sanguinis*, indicating that the applied concentrations lacked bactericidal activity.	[3]
Galarraga-Vinueza, M.E. et al. (2020) A-type proanthocyanidins	*In vitro*	To assess the effects of various Cranberry concentrates on cell viability, anti-inflammatory activity, and macrophage polarization.	AC-PACs did not reduce the viability of human gingival fibroblasts, human osteosarcoma, or macrophages. Cranberry downregulated pro-inflammatory cytokine expression and upregulates the expression and upregulated IL-10 expression. Macrophages stimulated with lipopolysaccharide and exposed to Cranberry showed decreased M1 polarization and increased M2 polarization.	[63]
Singhal, R. et al. (2020) Cranberry extract	*In vitro*	To analyze the antimicrobial and antibiofilm effects of Cranberry against *Streptococcus mutans* and *Lactobacillus acidophilus*.	Cranberry demonstrated bactericidal, bacteriostatic, and anti-biofilm activities against *Streptococcus mutans* and *Lactobacillus acidophilus* in a time- and dose-dependent manner.	[64]
Abu-obaid, E. et al. (2020) Cranberry extract (*0.3%)* mixed with chlorhexidine digluconate (0.06%)	*In vitro*	To assess the antimicrobial activity of natural and semi-natural mouthrinses against *Streptococcus mutans*, *Lactobacillus fermentum*, and *Lactobacillus casei* (isolated from the saliva samples) and their respective reference strains.	Cranberry combined with chlorhexidine demonstrated greater bacterial control than chlorhexidine with alcohol. Both the herbal mix and Cranberry mouthrinse showed potential as effective natural alternatives to chlorhexidine mouthrinse, with or without alcohol, for oral health promotion.	[65]
Kranz, S. et al. (2020) Cranberry juice	*In vitro*	To assess the antibacterial activity of blackcurrant, redcurrant, Cranberry, and raspberry juices against *Streptococcus mutans*, *Streptococcus gordonii*, *Streptococcus sobrinus*, *Actinomyces naeslundii*, *Fusobacterium nucleatum*, *Aggregatibacter actinomycetemcomitans*, *Porphyromonas gingivalis*, and *Enterococcus faecalis*.	Blackcurrant juice demonstrated the highest antibacterial efficacy, followed by redcurrant and Cranberry juice. Cranberry juice inhibited *Fusobacterium nucleatum* and *Aggregatibacter actinomycetemcomitans* to a degree similar to chlorhexidine and showed greater effectiveness in controlling *Streptococcus sobrinus* than chlorhexidine.	[66]
Ankola, A. et al. (2020) Cranberry extract	*In vitro*	Assess the cytotoxic potential of Cranberry extract on oral cancer KB cells.	Cranberry showed an antiproliferative effect on KB cell line and exhibited no cytotoxicity on the normal fibroblast cell line.	[6]
Keshaav Krishnaa, P.; Prabakar, J. (2020) Cranberry extract	*In vitro*	To analyze Cranberry extract distillate as a root canal irrigant.	Cranberry extract distillate demonstrated potent antimicrobial activities.	[67]
Islam, M.S. et al. (2020) Cranberry juice	*In vitro*	To evaluate the inhibitory effect of fresh Cranberry, strawberry, and blueberry juices on the viability of *Streptococcus mutans*, *Streptococcus pyogenes*, and *Streptococcus viridans*.	Cranberry juice had an antibacterial effect on *Streptococcus mutans*, *Streptococcus pyogenes*, and *Streptococcus viridans.*	[68]
Islam, M.S. et al. (2021) Cranberry juice	*In vitro*	To analyze Cranberry juice’s effects on demineralized dentin collagen, comparing its efficacy to glutaraldehyde.	Cranberry juice showed statistically significant effects on water sorption, ultimate tensile strength, and amount of collagen degradation compared to glutaraldehyde (control group).	[69]
Wu, C.D. et al. (2021) Cranberry extract	*In vitro*	To evaluate whether commercially available plant polyphenol-containing beverages may inhibit the growth and biofilm formation of *Streptococcus mutans* and dental plaque in children.	Cranberry juice inhibited the growth and biofilm formation of *Streptococcus mutans* and children’s supragingival plaque bacteria by over 90%. Plaque biofilms developed in the presence of Cranberry juice were loosely attached and easily removed from surfaces.	[70]
Souissi, M. et al. (2021) Polyphenolic fraction of blueberry, Cranberry, and strawberry (Orophenol^®^, Quebec, Canada)	*In vitro*	To assess the antibacterial, anti-biofilm, and anti-adhesion properties of a berry polyphenolic fraction (wild blueberry, Cranberry, and strawberry), marketed as Orophenol^®^, against *Streptococcus mutans*.	Orophenol^®^ inhibited *Streptococcus mutans* biofilm formation and adhesion to saliva-coated hydroxyapatite and saliva-coated nickel–chrome alloy in a dose-dependent manner. The berry fraction exhibited no cytotoxicity in an oral epithelial cell model.	[71]
Pellerin, G. et al. (2021) Cranberry extract	*In vitro*	To evaluate the impact of partially removing organic acids (0%, 19%, 42%, 60%, and 79%) from Cranberry juice using electrodialysis with a bipolar membrane on its antibacterial activity against periodontopathogenic bacteria and its anti-inflammatory effects in an oral epithelial cell model.	Cranberry juice enhanced the adherence of *Aggregatibacter actinomycetemcomitans* and *Porphyromonas gingivalis* to oral epithelial cells, while reducing the adherence of *Fusobacterium nucleatum*. Exposure of *Fusobacterium nucleatum* to Cranberry juice deacidified by ≥42% increased hydrogen sulfide production compared with the unmodified beverage. Removal of organic acids from Cranberry juice decreased its cytotoxicity toward oral epithelial cells. Additionally, IL-6 secretion by LPS-stimulated oral epithelial cells exposed to Cranberry juice rose proportionally with the degree of deacidification.	[72]
Wang, Y. et al. (2021) Cranberry extract	*In vitro*	To assess the effects of natural extracts and a chemical cross-linker on dentin collagen cross-linking, resistance to enzymatic degradation, and endogenous MMP activity under clinically relevant conditions.	Dentin collagen treated with Cranberry juice extract shows a rapid increase in resistance to enzymatic degradation and inhibition of MMP. A 30 s application of Cranberry juice extract may provide a clinically feasible method to enhance the longevity of dentin bonding in composite restorations.	[73]
Rahman, H. et al. (2021) Cranberry extract	*In vitro*	To determine the antioxidant activity of Cranberry extract, green tea, aloe vera, and sodium ascorbate, and their effects on restoring bond strength in bleached enamel surface.	All substances restored the reduced bond strength following bleaching, with green tea showing the greatest effect, followed by aloe vera, sodium ascorbate, and Cranberry.	[74]
Niemeyer, S. et al. (2021) Cranberry extract	*In vitro*	To analyze the modification of the salivary pellicle by various polyphenol-rich teas and natural extracts for dental erosion protection.	Greater demineralization was induced by Cranberry due to its low pH, resulting in the lowest surface microhardness and surface reflection intensity.	[75]
Pellerin, G. et al. (2021) Cranberry juice	*In vitro*	To evaluate the effects of Cranberry juice deacidified by electrodialysis with a bipolar membrane on its antibacterial activity against *Streptococcus mutans*, *Streptococcus sobrinus*, *Streptococcus gordonii*, *Streptococcus oralis*, and *Streptococcus salivarius*, as well as on oral epithelial barrier function and inflammatory response.	Removal of organic acids from Cranberry juice decreased its bactericidal activity against *Streptococcus mutans* and *Streptococcus gordonii*. Conditioning saliva-coated hydroxyapatite with Cranberry juice reduced the adherence of *Streptococcus mutans*, *Streptococcus sobrinus*, and *Streptococcus oralis.* Deacidified Cranberry juice preserved the integrity of a keratinocyte monolayer, although it increased IL-6 secretion without affecting IL-8 production by oral epithelial cells.	[76]
Anitha, K.V. et al. (2022) Cranberry extract incorporated into polymer of polymethyl methacrylate	*In vitro*	To evaluate the effectiveness of Cranberry extract in inhibiting *Candida albicans* adhesion to denture base biomaterials biofunctionalized with Cranberry.	Enrichment of self-curing polymethyl methacrylate with Cranberry extract reduced *Candida albicans* attachment. Cranberry also inhibited *Candida albicans* growth compared with immersion in distilled water.	[5]
Kato, M. et al. (2022) Cranberry juice and Cranberry extract	*In vitro*	To assess the Cranberry effect on dentin erosion.	All treatments (green tea extract solution containing 400 µm EGCG, 10% Cranberry extract, and Cranberry juice) showed a significant reduction in dentin wear compared with the control (distilled water). No significant differences were observed among the treatments, despite the acidic pH of the solutions.	[7]
Vaillancourt, K. et al. (2022) Combination of Cranberry, blueberry, and strawberry extracts (Orophenol^®^)	*In vitro*	To determine the impact of Cranberry, blueberry, and strawberry extracts (Orophenol^®^) on *Porphyromonas gingivalis*.	The berry polyphenolic fraction (Orophenol^®^) diminished several pathogenic traits of *Porphyromonas gingivalis*. It inhibited bacterial growth and decreased hemolytic activity, adherence to a basement membrane matrix model, proteolytic enzyme activity, and the generation of reactive oxygen species by oral keratinocytes.	[77]
Wang, R. et al. (2022) A-type linkage proanthocyanidins from Cranberry extract	*In vitro*	To evaluate dentin collagen stabilization effects of three flavonoids from natural extracts: A-PA, B-PA, and EGCG.	A-PA and B-PA provided superior collagen stabilization compared with EGCG at concentrations of 0.65% and 1.3% (*p* < 0.01).	[78]
Alanazi, A.M. et al. (2023) Cranberry solution	*In vitro*	To investigate the effect of bleaching methods (40% hydrogen peroxide and ZP) PDT-activated with the utilization of different reversal procedures (10% ascorbic acid and 6% Cranberry solution) on bonding, surface microhardness, and surface roughness of the enamel surface.	Enamel surface bleached with 40% hydrogen peroxide and reversed using 10% ascorbic acid exhibited the highest shear bond strength, whereas 40% hydrogen peroxide without a reversal agent showed the least shear bond strength. On surface microhardness, enamel treated with ZP activated by PDT and reversed with 10% ascorbic acid demonstrated the highest surface microhardness, while bleaching with 40% hydrogen peroxide followed by reversal with 6% Cranberry solution resulted in the lowest surface microhardness. Regarding surface roughness, samples bleached with 40% hydrogen peroxide and reversed with 6% Cranberry solution showed the highest values, whereas enamel bleached with ZP activated by PDT and reversed with 6% Cranberry exhibited the lowest surface roughness.	[79]
Baumann, T. et al. (2023) Polyphenols from Cranberry extract	*In vitro*	To formulate a rinsing solution enriched with natural polyphenol-rich extracts, derived from grapeseed or Cranberry, aimed at preventing dental erosion.	Polyphenol solutions containing fluoride and additional flavors provided greater enamel protection than fluoride alone, and performed similarly to the Sn^2+^/F- solution in terms of relative surface microhardness and calcium release. In surface reflection intensity measurements, Sn^2+^/F- offered the highest protection, while the polyphenol solutions showed results comparable to fluoride alone.	[80]
Nisar, S. et al. (2023) Type-A proanthocyanidins from Cranberry juice	*In vitro*	To investigate the capacity of various crosslinkers to enhance or restore the properties of denatured dentin collagen.	Compared with control, cross-linking induced by theaflavins from black tea and AC-PACs from Cranberry juice significantly enhanced collagen biostability (reduced weight loss and hydroxyproline release, *p* < 0.05), inhibited endogenous MMP (*p* < 0.001), and improved mechanical properties (*p* < 0.05), irrespective of collagen denaturation.	[81]
Silva, A.M. et al. (2023) Cranberry solution 6%	*In vitro*	To assess the effects of antioxidant solutions on fracture strength and bonding performance in non-vital teeth treated with 38% hydrogen peroxide bleaching.	The tested antioxidants (10% sodium ascorbate, 10% alpha-tocopherol, 5% Cranberry, or 0.0025% capsaicin) had no significant effect on fracture strength, hybrid layer thickness, or dentin bond strength following bleaching endodontically treated teeth.	[82]
Bauer, Y. et al. (2024) Cranberry extract	*In vitro*	To evaluate Cranberry extract’s potential to promote osteogenesis.	Cranberry concentrations (ranging from 62.5 to 500 mg/mL) were biocompatible with osteoblasts and mesenchymal stromal cells. At 20 mg/mL, Cranberry enhanced ALP activity by 2-fold and increased BMP-2 expression by nearly 1.5-fold compared to the positive control (osteogenic medium: DMEM supplemented with FBS, 50 μM L-ascorbic acid, and 10 mM β-glycerophosphate). A concentration of 200 mg/mL stimulated a 1.7-fold increase in extracellular matrix mineralization relative to the positive control.	[83]
Dame-Teixeira, N. et al. (2024) Cranberry extract	*In vitro*	To develop a dysbiotic root caries biofilm model for studying microbial modulation, and to evaluate the effects of natural substances (phenolic lipid from cashew nutshell liquid and Cranberry extract) applied during biofilm formation or on mature biofilms.	Root pre-treatment with Cranberry reduced microbial viability and gelatinase activity, while collagenase activity remained unaffected (*p* < 0.05).	[84]
Ingle, A.S. et al. (2024) Cranberry extract	*In vitro*	To analyze the synergistic effects of CPP-ACPF combined with plant-derived dentin biomodifying agents (grape seed, green tea, and Cranberry extracts) on the biomimetic remineralization of eroded dentin.	A synergistic effect was observed when CPP-ACPF was applied following pre-treatment with plant-derived dentin biomodifying agents, enhancing biomimetic remineralization of eroded dentin.	[85]
Lewis, N.V. et al. (2024) Cranberry extract	*In vitro*	To determine the degree of conversion of an 8th-generation bonding agent in sound versus caries-affected dentin following pre-treatment with Cranberry or mulberry extracts in combination with MMP inhibitors.	Pre-treatment of the dentin with MMP inhibitors increased the degree of conversion in sound dentin, but not in caries-affected dentin. The highest degree of conversion was observed in sound dentin samples pre-treated with mulberry extract.	[86]
Shetty, P. et al. (2024) Cranberry juice	*In vitro*	To assess the effect of different solutions (artificial saliva, Biotene, orange juice, passion fruit juice, Sprite, Coca-Cola, apple cider vinegar, and Cranberry juice) on the color stability and surface hardness of a nanohybrid dental composite in simulated oral conditions.	Polished surfaces exhibited a 21.9–35.5% decrease in microhardness after 28 days, with apple cider vinegar and Cranberry juice producing the greatest reductions. In contrast, non-polished surfaces showed an 11.2–17.4% increase in microhardness. Color changes were more pronounced on polished surfaces, with Coca-Cola and Cranberry juice causing the largest differences.	[87]
Adami, G.R. et al. (2025) Cranberry juice	*In vitro*	To demonstrate that shed oral biofilms (obtained from donor saliva and tested under optimized conditions) respond consistently to antibacterial challenges, as indicated by reductions in rRNA accumulation in susceptible bacterial taxa.	Cranberry juice inhibited multiple oral taxa, including *Alloprevotella species*, *Granulicatella adiacens*, *Lachnoanaerobaculum umeaense*, *Lepotrichia species*, *Peptostreptococcus stomatis*, *Prevotella nanceiensis*, *Stomatobaculum species*, and *Veillonella parvula*, and eliminated certain susceptible targets.	[88]
Mailart, M.C. et al. (2025) Cranberry extract	*In vitro*	To evaluate the impacts of modifying the salivary pellicle with polyphenol-rich solutions containing fluoride on enamel erosion and abrasion.	The greatest surface loss was observed in the deionized water group (*p* < 0.001). All other tested solutions (including grape seed extract +500 ppm F^−^, Cranberry extract +500 ppm F^−^, 500 ppm F^−^ sodium fluoride solution, a commercial solution, SnCl_2_/NaF/AmF) provided significant protection against erosive-abrasive challenges, with no differences among solutions.	[89]
Nisar, S. et al. (2025) Cranberry extract	*In vitro*	To develop crosslinker-modified etchants using phosphoric acid and an organic acid mixture (35% tartaric acid and 10% PA) supplemented with 1% theaflavins, Cranberry extract, or EDC/NHS, aiming for effective dentin demineralization while improving solubility, and to evaluate their effects on bond strength, nanoleakage, and MMP activity in sound and caries-affected dentin before and after thermocycling.	Crosslinker-modified etchants, especially those containing theaflavins and Cranberry extract, offered a promising strategy for simultaneous dentin etching and biomodification, improving bonding durability on clinically relevant substrates.	[90]
Viswanathan, A.K. et al. (2025) Cranberry extract	*In vitro*	To investigate the surface microhardness, flexural, and impact strength of heat-activated polymethyl methacrylate denture base resin reinforced with different concentrations of Cranberry extract.	Incorporating up to 2 wt.% Cranberry into heat-activated polymethyl methacrylate enhanced the surface microhardness, flexural, and impact resistance compared to 0 wt.% control.	[91]

**Legend:** AC-PACs—A-type Cranberry proanthocyanidins; AGEs—Advanced glycation end products; ALP—alkaline phosphatase; AP-1—Activator protein-1 transcription factor; A-PA—A-type linkage proanthocyanidins; BMP-2—bone morphogenetic protein 2; B-PA—B-type linkage proanthocyanidins; c-Jun—c-Jun component (core protein that forms the AP-1 complex); CPP-ACPF—Casein phosphopeptide amorphous calcium phosphate fluoride; DMSO—Dimethyl sulfoxide; EDC/NHS—1-ethyl-3-(3-dimethylaminopropyl)carbodiimide and N-hydroxysuccinimide; EGCG—epigallocatechin-3-gallate; ERK1/2—Extracellular signal-regulated kinase; F-ATPases—F-Type ATPase; FTF—Fructosyltransferase; 1/2; G-CFS—Granulocyte Colony-Stimulating Factor; G-HSA—Glycated human serum albumin; GRO-α—Growth regulated oncogene alpha; GTF—Glucosyltransferase; IκBα—Inhibitor kappa B alpha; IL-1β—Interleukin-1 beta; IL-6—Interleukin 6; IL-8—Interleukin 8; IL-10—Interleukin 10; IL-17—Interleukin 17; IP-10—Interferon-gamma-inducible protein 10; LPS—lipopolysaccharides; LtxA—Leukotoxin; MAPK/AP-1—Mitogen-Activated Protein Kinase/Activator Protein-1; MCP-1—Monocyte chemoattractant protein-1; MMP—matrix metalloproteinase; MMP1—matrix metalloproteinase-1; MMP-3—matrix metalloproteinase-3; MMP-9—matrix metalloproteinase-9; NDM—high-molecular-weight non-dialyzable material; NDMac—aqueous acetone-soluble fraction; NF-κB—Nuclear factor kappa-light-chain-enhancer of activated B cells; PA—phosphoric acid; PDT—Photodynamic therapy; PGE2—Prostaglandin E2; phospho-c-Jun—c-Jun protein phosphorylated by kinases; rRNA—Ribonucleic acid.; sHA—Saliva-coated hydroxyapatite; TMJ—Temporomandibular Joint; VEGF—Vascular Endothelial Growth Factor; ZP—Zinc Phthalocyanine.

**Table 3:** Summary of clinical and hybrid (*in vitro* and clinical) included studies’ descriptive characteristics in chronological order.

Author (Year) Cranberry Fraction (*Vaccinium macrocarpon* Specie)	Type of Study	Aim	Outcomes	Reference
Weiss, E.I. et al. (2002) NDM	*In vitro Clinical trial*	To investigate the influence of the NDM from Cranberry juice on the coaggregation behavior of oral bacterial species.	NDM inhibited the coaggregation of oral bacteria and their adhesion to epithelial cells. Additionally, NDM altered the composition of the salivary microbiota in humans.	[92]
Weiss, E.I. et al. (2004) NDM	*In vitro* *Clinical trial*	To investigate how a mouthwash formulated with NDM influences salivary bacterial counts in healthy individuals.	NDM inhibited the adhesion of *Streptococcus sobrinus* to saliva-coated hydroxyapatite *in vitro* and reduced the total salivary bacterial load. However, NDM did not affect gingival or plaque indices.	[93]
Gupta, A. et al. (2015) NDM	*Clinical trial*	To comprehend the effect of the NDM-containing mouthrinse on *Streptococcus mutans* colonization in children.	NDM mouthrinse induced a reduction in *Streptococcus mutans* after 30 days compared with a control mouthrinse lacking a high-molecular-weight component.	[94]
Khairnar, M. et al. (2015) Cranberry extract	*Clinical trial*	To determine the relative efficacy of mouthwashes containing chlorhexidine or Cranberry on *Streptococcus mutans*.	Cranberry mouthwash demonstrated comparable efficacy to chlorhexidine mouthwash, suggesting it as a viable alternative.	[95]
Woźniewicz, M. et al. (2018) Cranberry functional beverage [Apple juice (80% *v/v*) with Cranberry juice (20% *v/v*), and grounded cinnamon (0.25 g)]	*Clinical* *trial*	To explore the ability of Cranberry functional beverage to reduce gingival inflammation through inhibition of dental biofilm formation, and to assess potential changes in antioxidant status and systemic inflammation in individuals with gingivitis.	Cranberry functional beverage demonstrated reductions in gingival inflammation, plaque index, and approximal plaque index compared with the control group (water).	[96]
Zare, A.J. et al. (2018) Cranberry juice and Cranberry juice enriched with omega-3	*Clinical trial*	To test whether Cranberry juice enriched with omega-3 may improve glycemic control, lipid profile, and periodontal status in diabetic patients with periodontal disease.	Cranberry juice enriched with omega-3 consumption reduced glycated hemoglobin, increased HDL-C, and improved periodontal conditions. Consequently, this Cranberry juice was suggested as an adjunct to nonsurgical periodontal therapy.	[97]
Philip, N. et al. (2020) Casein phosphopeptide–amorphous calcium phosphate dentifrice supplemented with a polyphenol-rich Cranberry extract	*Clinical trial*	To clinically evaluate the microbial effects of two dentifrices: one containing casein phosphopeptide–amorphous calcium phosphate and the other supplemented with Cranberry extract.	The dentifrice containing Cranberry reduced the abundance of caries-associated bacteria and increased the proportion of health-associated bacteria, with the exception of *Corynebacterium durum*.	[98]
Kharche, A. et al. (2021) Cranberry extract	*Clinical trial*	To investigate the antimicrobial properties and the efficacy of different herbal mouthrinses on orthodontic patients diagnosed with gingivitis.	All phytotherapeutic agents effectively reduced plaque and gingivitis scores in individuals with fixed orthodontic treatment. Cranberries may serve as a useful adjunct to non-surgical periodontal therapy.	[99]
Laky, B. et al. (2023) Multinutrient supplement containing Cranberry extract	*Clinical trial*	To clinically analyze the efficacy of a multinutrient supplement as an adjunctive therapy to scaling and root planning in periodontitis patients.	Multinutrient supplementation led to a significantly greater reduction in pocket depth probing (-0.75 ± 0.42 mm) and bleeding on probing (-21.9 ± 16.1%) from baseline to re-evaluation compared with placebo (-0.51 ± 0.30 mm, *p* = 0.040 and -12.5 ± 9.8%, *p* = 0.046, respectively). Improvements in all other periodontal parameters were higher in the supplement group than in the placebo group, but these differences were not statistically significant (*p* > 0.05).	[100]
Bansal, K. et al. (2024) NDM	*Clinical trial*	To explore the effectiveness of the NDM-containing mouthrinse on *Streptococcus mutans* counts and compare it with a sodium fluoride mouthrinse.	The NDM-containing mouthrinse was shown to be non-inferior to the fluoride mouthrinse regarding changes in Streptococcus mutans counts.	[101]
Figueiredo, L.C. et al. (2024) DESPLAC^®^ gel (São Paulo, Brazil)-containing propolis, aloe vera, green tea, Cranberry, and calendula	*Clinical trial*	To clinically assess the effects of a nature-based gel (containing propolis, aloe vera, green tea, Cranberry, and calendula) on gingival inflammation control and its impact on the proteomic profile of gingival crevicular fluid.	The nature-based gel produced clinical outcomes comparable to conventional dentifrices. However, it significantly altered the proteomic profile of gingival crevicular fluid after treatment, reflecting a host-response-associated profile.	[102]
Agrawal, A. et al. (2025) Cranberry extract	*Clinical trial*	To clinically analyze the efficacy of mouthwashes containing Cranberry extract or fluoride to decrease *Streptococcus mutans* counts in children with moderate-to-severe dental caries.	Cranberry extract and fluoride mouthwashes produced statistically significant reductions in *Streptococcus mutans* colony-forming units at 3 months (*p* < 0.01) and 6 months (*p* < 0.01) in relation to baseline.	[103]
Olczak-Kowalczyk, D. et al. (2025) Cranberry extract	*Clinical trial*	To clinically assess the effect of a tablet containing inactivated *Lactobacillus salivarius* and Cranberry extract on caries development in preschool children with active caries.	Daily consumption of a tablet containing a paraprobiotic and Cranberry extract decreased the 9-month incidence of initial non-cavitated carious lesions in caries-active preschool children.	[104]

**Legend:** HDL-C—High-density lipoprotein cholesterol; MMP-9—matrix metalloproteinase-9; NDM—high-molecular-weight non-dialyzable material; NDMac—aqueous acetone-soluble fraction.

## Data Availability

The original contributions presented in this study are included in the article. Further inquiries can be directed to the corresponding author(s).
